# Effects of the clathrin inhibitor Pitstop-2 on synaptic vesicle recycling at a central synapse *in vivo*

**DOI:** 10.3389/fnsyn.2022.1056308

**Published:** 2022-11-17

**Authors:** Alp Paksoy, Simone Hoppe, Yvette Dörflinger, Heinz Horstmann, Kurt Sätzler, Christoph Körber

**Affiliations:** ^1^Department of Functional Neuroanatomy, Institute of Anatomy and Cell Biology, Heidelberg University, Heidelberg, Germany; ^2^School of Biomedical Sciences, University of Ulster, Coleraine, United Kingdom

**Keywords:** 3D reconstruction, calyx of Held synapse, clathrin, electron microscopy, synaptic vesicle cycle

## Abstract

Four modes of endocytosis and subsequent synaptic vesicle (SV) recycling have been described at the presynapse to ensure the availability of SVs for synaptic release. However, it is unclear to what extend these modes operate under physiological activity patterns *in vivo*. The coat protein clathrin can regenerate SVs either directly from the plasma membrane (PM) via clathrin-mediated endocytosis (CME), or indirectly from synaptic endosomes by SV budding. Here, we examined the role of clathrin in SV recycling under physiological conditions by applying the clathrin inhibitor Pitstop-2 to the calyx of Held, a synapse optimized for high frequency synaptic transmission in the auditory brainstem, *in vivo.* The effects of clathrin-inhibition on SV recycling were investigated by serial sectioning scanning electron microscopy (S^3^EM) and 3D reconstructions of endocytic structures labeled by the endocytosis marker horseradish peroxidase (HRP). We observed large endosomal compartments as well as HRP-filled, black SVs (bSVs) that have been recently recycled. The application of Pitstop-2 led to reduced bSV but not large endosome density, increased volumes of large endosomes and shifts in the localization of both types of endocytic compartments within the synapse. These changes after perturbation of clathrin function suggest that clathrin plays a role in SV recycling from both, the PM and large endosomes, under physiological activity patterns, *in vivo*.

## Introduction

Local SV recycling at the presynapse is an essential process for the maintenance of neurotransmission (Ceccarelli et al., 1973; Heuser and Reese, 1973). However, the exact molecular mechanisms underlying SV recycling are still not completely understood, especially *in vivo* (for review see Kokotos and Cousin, 2015; Watanabe and Boucrot, 2017; Chanaday et al., 2019; Ivanova and Cousin, 2022). Till date, four different, mutually non-exclusive mechanisms for synaptic endocytosis have been described. CME retrieves individual SVs with the help of a clathrin coat directly from the PM (Heuser and Reese, 1973; Murthy and De Camilli, 2003; Logiudice et al., 2009). During activity dependent bulk endocytosis (ADBE), a large piece of presynaptic membrane is taken-up from the PM to form an endosome from which SVs can bud (e.g., Miller and Heuser, 1984; Holt et al., 2003; Wu and Wu, 2007; Kokotos and Cousin, 2015). Ultrafast endocytosis (UFE) retrieves a portion of the PM equivalent to 4 SVs, which is much less than ADBE (Watanabe et al., 2013, 2014). During kiss-and-run, a fusion pore is transiently opened without full collapse of the SV into the PM. This allows the retrieval of the SV as a whole (Gandhi and Stevens, 2003; He et al., 2006; Zhang et al., 2009). Different endocytosis modes have been linked to distinct levels of activity; CME and UFE for example are thought to operate at low to moderate synaptic activity levels, while ADBE is suggested to be employed only during periods of high synaptic activity (Balaji and Ryan, 2007; Zhu et al., 2009; Leitz and Kavalali, 2011, 2014; Watanabe et al., 2013; Delvendahl et al., 2016; Chanaday and Kavalali, 2018). However, it is not clear which of these mechanism(s) is operating *in vivo*, under physiological activity patterns, as most studies have been conducted in cultured neurons or brain slices (e.g., de Lange et al., 2003; Granseth et al., 2006; Kim and Ryan, 2009; Watanabe et al., 2013, 2014; Soykan et al., 2017). We therefore sought out to examine endocytosis *in vivo* in a synapse optimized for high frequency synaptic transmission.

A prime model synapse operating at high frequencies is the calyx of Held, a giant, glutamatergic, axosomatic synapse formed between the globular bushy cells of the contralateral anterior ventral cochlear nucleus and the principal cells of the medial nucleus of the trapezoid body (MNTB) in the auditory brainstem. The calyx of hearing animals can faithfully transmit signals at frequencies of several hundred Hertz but has to undergo extensive structural and functional remodeling around the onset of hearing, in order to meet the requirements of processing airborne sounds (Borst and Soria van Hoeve, 2012). Two different forms of endocytic compartments have been identified at the calyx using the endocytosis marker HRP: large tubular or cisternal endosomes and recently recycled SVs (termed bSVs from here on) (de Lange et al., 2003; Körber et al., 2012), although their origin, in particular which endocytosis mechanism(s) has been operational, remained elusive.

To gain further insights into the mechanisms of SV recycling employed during physiological, high frequency synaptic transmission, we examined the role of clathrin at the calyx of Held *in vivo*. Therefore, we applied the clathrin inhibitor Pitstop-2 (von Kleist et al., 2011) alongside with HRP to the MNTB of early hearing rats just after the onset of hearing, a time at which the mature pattern of signal transmission has been established at the calyx (Crins et al., 2011; Sonntag et al., 2011). The rats were subjected to environmental noise inducing physiological synaptic transmission patterns at the calyx. Synaptic endocytosis in response to this stimulation was examined by electron microscopy and 3D analysis of the size and distribution of HRP-filled bSVs and large endosomes. Application of Pitstop-2 led to a decrease in bSV density, an increased volume of large endosomes and a shift in bSV and large endosome localization toward the presynaptic membrane.

## Materials and methods

### Stereotaxic injections

All experiments were conducted in accordance with the German federal law and the EU directive 2010/63. The protocols were approved by the local authority (Regierungspräsidium Karlsruhe). Sprague Dawley rats of either sex were injected at P12/13 with either HRP only (10 mg/ml in PBS, SERVA, Heidelberg, Germany), HRP supplemented with 1% DMSO or HRP along with Pitstop-2 (120 μM, Sigma) into the MNTB as described previously (Körber et al., 2012). In brief, rats were anesthetized, transferred into a non-traumatic stereotaxic frame (Kopf Instruments, Tujunga, CA) and 2 μl of marker or drug solution were injected to the following coordinates relative to bregma and midline (x, y, z in mm): 0.95, –6.4, –7.6. Pitstop-2 was delivered in a two-step injection. First, Pitstop-2 was administered to the MNTB alone and only in the second injection, 15 min after the first one, Pitstop-2 was co-injected with HRP to the identical coordinates. The rats remained anesthetized between the two injections. After surgery, rats recovered quickly and behaved normally while they were exposed to standard laboratory environmental noise (radio, air conditioner, human conversation) for 30 min.

### Fixation and tissue processing

Rats were deeply anesthetized and transcardially perfused with 15 ml of PBS followed by 15 ml PFA (4% in PBS). The brain was removed and post-fixed at 4°C over night. The brainstem was cut into 100 μm thick sections including the MNTB on a vibratome (Sigmann Elektronik, Hüffenhardt, Germany). The sections were prepared for the DAB reaction as described previously (Körber et al., 2012). In brief, the sections were incubated in α-D-glucose (2 mg/ml) and 3,3′diaminobenzidine (DAB) (1.4 mg/ml) dissolved in PBS for 20 min in the dark. *Aspergillus* glucose oxidase (0.1 mg/ml, SERVA) was added to start the DAB polymerization. The DAB reaction was stopped after 1 h by incubating the sections in cacodylic acid (100 mM) for 30 min. The MNTB was excised and post-fixed for 1 h in 1.5% potassium-ferry-cyanide and 2% osmium tetroxide on ice in the dark. The samples were rinsed three times in distilled water, dehydrated in an ascending series of ethanol, incubated in epoxy/propylenoxide (1:1) over night and embedded in epoxy resin, which was polymerized at 60°C for 36 h.

### Serial section scanning electron microscopy

Serial section scanning electron microscopy (S^3^EM) was performed as described before (Horstmann et al., 2012). Ribbons of 20 serial sections (40 nm) were cut through the MNTB using an Ultracut S ultramicrotome (Leica) equipped with a diamond knife angled at 45° (Diatome, Biel, Switzerland). Sections were collected on clean silicon wafers (Si-Mat Silicon Materials, Landsberg, Germany) that were glow discharged for 30 s right before tissue sectioning. Tissue compression due to sectioning was neutralized by exposure to chloroform vapor. The samples were dried and stained following a modified Reynolds-procedure [saturated uranyl acetate solution (16 min) followed by lead citrate (8 min)]. Scanning electron microscopy was performed using a LEO Gemini 1530 equipped with a field emission gun and an ATLAS scanning generator (Zeiss) using the InLens detector at the following settings: 3.6 mm working distance, 30 μm aperture and 2 keV acceleration voltage. Images of 2632 × 2632 pixels were taken at a pixel size of 3.8 nm (dwell time 25.6 μs).

### Data analysis

Images were only taken from tissue that did not show signs of mechanical damage due to the injection procedure. Calyces were randomly chosen within the MNTB. However, since continuous supply of HRP during the loading period is crucial for the study of endocytosis, only calyces fully surrounded by HRP were considered for further analysis. 18-22 consecutive EM sections were manually aligned according to prominent structures present in consecutive sections for 3D reconstruction using OpenCAR software (Sätzler et al., 2002). The PM, the innervation side and the large endosomal compartments were manually contoured in each aligned section. In order to evade artifacts intrinsic to the imaging process, only large endosomes spanning no less than two consecutive sections (80 nm) were considered for 3D reconstruction. bSVs were identified by their round morphology, uniform black labeling and SV-like diameter. We controlled for variability in the DAB reaction by measuring intensity profiles of randomly chosen bSVs. bSVs were only considered for further analysis if intensity values were homogeneous along the profile (as opposed to the two distinct intensity peaks observed at the membrane of non-labeled SVs) and at least 1.5-fold higher than those of non-labeled SV membrane peaks. bSVs were reconstructed as spheres based on their diameter (Sätzler et al., 2002). 3D reconstructions were performed following the Delaunay method (Boissonnat and Geiger, 1992). OpenCARnEval, a command-line version of OpenCAR, provided numerical readouts of the structural features using batch scripts.

### Statistics

The population means and standard deviations (SD) of each experimental group were estimated by equal weighing of the averages and SDs of the individual synapses within the group as described previously (Körber et al., 2012). Utilizing these normal population estimates, we analyzed the two population means according to their differences and similarities by establishing the 95% confidence interval for the differences between two group means of normally distributed data (Berry, 1995). Since volumes of large endosomes were not normally distributed, they were first log transformed and differences in large endosome volume were calculated from the transformed data. The positions of large endosome and bSVs within the synapse were determined by classifying them as either closer to the innervation side or to the backside of the synapse (Körber et al., 2012). We compared the proportions of large endosomes or bSVs that were closer to either side by approximating the measured β-density of proportions in each experiment via a normal distribution (Berry, 1995). These approximations were used to determine the corresponding confidence intervals as described above. Data is represented as mean ± SEM. Significance in object densities, cell average-based endosome volumes and cell average-based distances was assigned by one-way ANOVA or nested one-way ANOVA as indicated, using Prism 9 software (GraphPad). Q-Q-plots were generated using R software. The experimental distributions obtained under the various conditions were compared to two simulated conditions assuming either identical distributions (Figure 5, red lines) or similar, not significantly different distributions (Figure 5, blue lines). The similar, not significantly different distributions were obtained by linear regression of the quantiles of the compared distributions.

**FIGURE 1:**
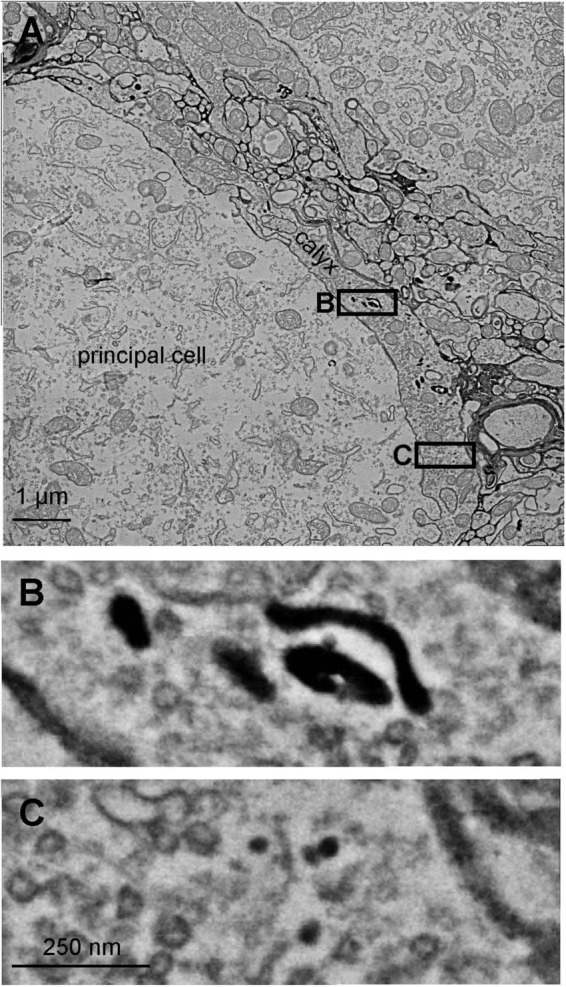
*In vivo* application of HRP labels endocytic structures at the calyx of Held synapse. **(A)** EM image of a P12 calyx of Held surrounded by HRP reaction product. Endocytic compartments are evident in the calyx as well as the MNTB principal cell. **(B,C)** Higher magnifications from the framed areas in **(A)** showing large endosomes **(B)** and bSVs **(C)**, respectively. Scale bar is 1 μm **(A)** or 250 nm **(B,C)**.

**FIGURE 2:**
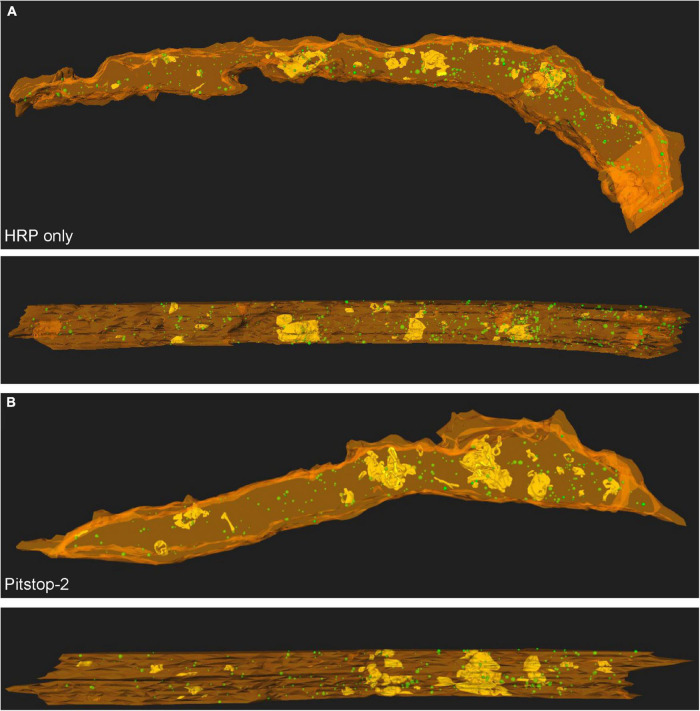
Large endosomes and recently recycled bSVs are located throughout the calyx volume, irrespecitve of the presence or absence of Pitstop-2. **(A)** 3D reconstructions of endocytic compartments in a calyx of Held segment at P12/13 in the absence of Pitstop-2 (HRP only control). **(B)** 3D reconstructions of endocytic compartments in a calyx of Held segment at P12/13 in the presence of Pitstop-2. PM appears in orange, large endosomes are depicted in yellow and bSVs as green spheres. The innervation side is facing the bottom (top) or the front (bottom).

**FIGURE 3:**
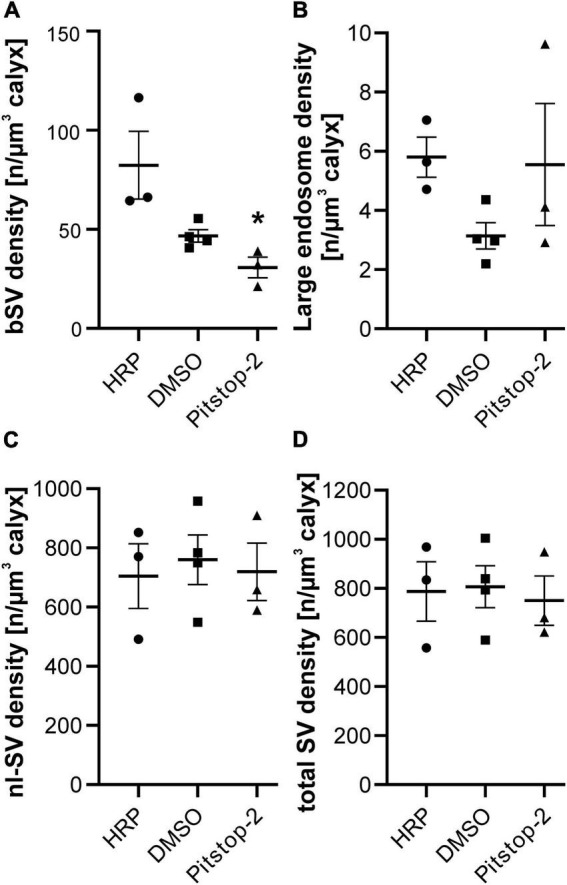
The density of large endosomes is unchanged after Pitstop-2 treatment while the density of bSVs decreases. **(A–D)** Quantification of the effects of Pitstop-2 on the density of bSVs **(A)**, large endosomes **(B)**, non-labeled SV (nl-SVs) **(C)** and total SVs (bSVs + nl-SVs) **(D)** at the calyx of Held [number of endosomes or SVs per μm^3^ calyx volume, *n* = 3–4 calyces/group, 1 calyx/animal; * = *p* < 0.05 one-way ANOVA followed by Dunnett’s *post hoc* test (HRP only vs. Pitstop-2)].

**FIGURE 4:**
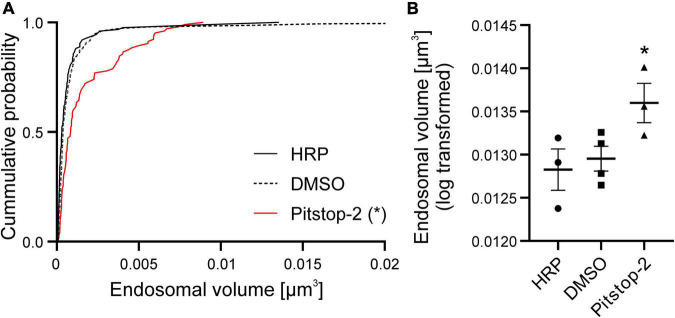
The volume of large endosomes is increased at the calyx of Held upon application of Pitstop-2. **(A)** Cumulative probability plot of all large endosome volumes observed in the presence and absence of Pitstop-2 (*n* = 104–152 endosomes from 3 to 4 calyces/group, 1 calyx/animal; * = *p* < 0.05, Pitstop-2 treatment significantly different from both controls). **(B)** Quantification of log transformed endosome volumes [*n* = 3–4 calyces/group, 1 calyx/animal; * = *p* < 0.05 nested one-way ANOVA followed by Dunnett’s *post hoc* test (HRP only vs. Pitstop-2)].

**FIGURE 5:**
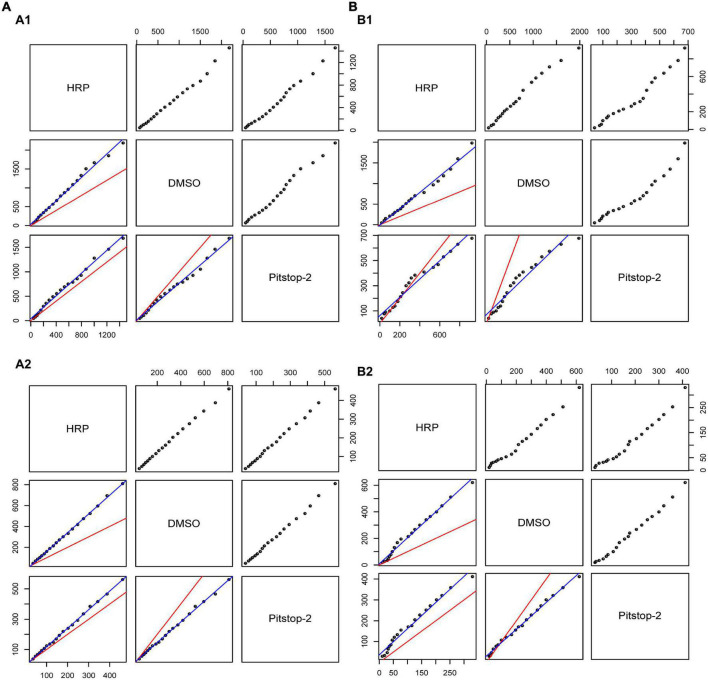
The distances between endocytic compartments and the PM at the innervation side or backside are insensitive to Pitstop-2 treatment. **(A,B)** Quantile-Quantile plots for probability distributions of mean distances from bSVs **(A)** and large endosomes **(B)** to the PM at the innervation side **(A1, B1)** and to the PM at the back of the synapse **(A2, B2)** in comparison between control and treatment groups. The red line represents the case of identical distributions, whereas the blue line represents similar, statistically not significantly different distributions (see Materials and methods) (*n* = 3–4 calyces/group, 1 calyx/animal).

## Results

The functional relevance of CME for SV recycling has recently been challenged (Kim and Ryan, 2009; Kononenko et al., 2014; Watanabe et al., 2014; Delvendahl et al., 2016; Soykan et al., 2017; Yu et al., 2018). We therefore examined the effect of the clathrin inhibitor Pitstop-2 (von Kleist et al., 2011) on synaptic endocytosis and SV recycling at the calyx of Held synapse *in vivo*. Pitstop-2 was applied together with HRP by stereotaxic injection into the MNTB of anesthetized rats. After recovery from anesthesia, the rats were subjected to environmental noise for 30 min, which provides physiological stimulation to the calyx of Held synapse. The properties of HRP filled presynaptic endocytic compartments, identified by the DAB reaction product, were examined by S^3^EM (Horstmann et al., 2012) (Figure 1). In order to examine the mechanisms of endocytosis active during high frequency synaptic activity induced by a physiological stimulus (airborne sound), we used early hearing rats at P12/13 that had already established the mature pattern of synaptic transmission at the calyx (Crins et al., 2011; Sonntag et al., 2011). Throughout the study, we compared the results of Pitstop-2 application to two independent control groups: rats injected only with HRP, and rats injected with HRP supplemented with 1% DMSO which was used to dissolve Pitstop-2.

### Pitstop-2 does not block synaptic vesicle recycling *in vivo*

The application of HRP *in vivo* resulted in the labeling of two forms of endocytic structures – large endosomes (Figure 1B) and bSVs (Figure 1C) (Körber et al., 2012) – irrespective of the presence of Pitstop-2 (Figure 2). Large endosomes were defined as HRP-labeled structures spanning at least two consecutive sections. Both types of endocytic compartments were detected throughout the calyx volume, in the vicinity of the innervation side as well as in areas toward the backside of the synapse (see below). The presence of bSVs, which have been generated either directly via CME or via budding from HRP-filled endosomes, suggests that SV recycling persists in the presence of Pitstop-2 although clathrin function is impaired (Figure 2B). Analysis of the densities of endocytic compartments, however, revealed a moderate decrease in bSV density after Pitstop-2 application (Pitstop-2: 30.73 ± 5.20 bSVs/μm^3^, HRP only: 82.33 ± 17.06 bSVs/μm^3^, HRP and DMSO: 46.69 ± 3.15 bSVs/μm^3^; *p* = 0.019, one-way ANOVA, Dunnett’s *post hoc* test: HRP only vs. HRP and DMSO: *p* > 0.05, HRP only vs. Pitstop-2: *p* = 0.013) (Figure 3A), which was not due to a sampling bias caused by differences in the reconstructed calyx volume (HRP only: 7.42 ± 1.19 μm^3^, HRP and DMSO: 11.62 ± 1.55 μm^3^, Pitstop-2: 5.96 ± 2.69 μm^3^; *p* = 0.14, one-way ANOVA). A block of SV recycling during ongoing synaptic transmission would eventually lead to the loss of SVs in the recycling pool and thus the mobilization and exocytosis of reserve pool SVs. Since these cannot be regenerated either under these conditions, block of SV recycling should eventually lead to a reduction in SV density in general. In order to assess such a loss in SV density, we quantified the density of non-labeled SVs (nl-SVs) in the reconstructed calyx segments, but did not observe a reduction upon Pitstop-2 application (HRP only: 705 ± 109 nl-SVs/μm^3^, HRP and DMSO: 760 ± 84 nl-SVs/μm^3^, Pitstop-2: 719 ± 97 nl-SVs/μm^3^; *p* = 0.91, one-way ANOVA) (Figure 3C). Moreover, the total SV density (sum of bSVs and nl-Sv per calyx volume) was unaffected by Pitstop-2 application (HRP only: 787 ± 121 SVs/μm^3^, HRP and DMSO: 807 ± 85 SVs/μm^3^, Pitstop-2: 750 ± 101 SVs/μm^3^; *p* = 0.92, one-way ANOVA) (Figure 3D). However, the total SV density is dominated by the density of nl-SVs, which is approximately 10 times higher than the bSV density, and thus occludes the moderate reduction in bSV density.

Despite the moderate decrease specifically in bSV density, we did not observe alterations in the density of large endosomes after Pitstop-2 application (Pitstop-2: 5.55 ± 2.06 endosomes/μm^3^, HRP only: 5.80 ± 0.68 endosomes/μm^3^, HRP and DMSO: 3.14 ± 0.45 endosomes/μm^3^; *p* = 0.9841, one-way ANOVA) (Figure 3B). Pitstop-2 application thus resulted in a moderate but specific reduction of the density of one type of endocytic compartments – bSVs – without effecting the density of the other type – large endosomes – in calyx of Held synapses.

### Pitstop-2 impairs the volume regulation of large endosomes

Although the density of large endosomes was unaffected by Pitstop-2 application, the volume of large endosomes was significantly increased in calyces treated with Pitstop-2 (measured values: Pitstop-2: 0.00145 ± 0.001 μm^3^ compared to 0.00084 ± 0.00104 μm^3^ and 0.00094 ± 0.00171 μm^3^ in the HRP only and HRP and DMSO groups, respectively, shown as cumulative probability plot; Figure 4A; *n* = 104–152 endosomes from 3 to 4 calyces/group, 1 calyx/animal; *d* = 0.73; *p* < 0.05 compared to both control groups based on confidence intervals of log transformed data; log transformed values: Pitstop-2: 0.01374 ± 0.00014 μm^3^, HRP only: 0.01277 ± 0.00009 μm^3^ HRP and DMSO: 0.01311 ± 0.00009 μm^3^; 3-4 calyces/group, 1 calyx/animal; *p* = 0.038 nested one-way ANOVA and Dunnett’s *post hoc* test, HRP only vs. Pitstop-2; Figure 4B). This increase in volume can be caused by two effects, which are not mutually exclusive. Either the application of Pitstop-2 induces a shift in the endocytosis mechanism toward clathrin-independent mechanisms such as ADBE and UFE due to impairment of CME at the PM, or the clathrin-dependent budding of bSVs from large endosomes is perturbed in the presence of Pitstop-2. Irrespective of the actual mechanism, these results show that clathrin is important for the size regulation of large (recycling) endosomes.

### Effects of Pitstop-2 on the localization of endocytic compartments

In addition to the density of endocytic compartments, we also investigated their localization within the synapse. This measure can provide insights into the location of endocytosis and/or the internal trafficking of recently endocytosed compartments. Therefore, we measured the distances from individual large endosomes and bSVs to the PM at the innervation side and at the back of the synapse. Although the back of the calyx seems to be an unlikely place for compensatory endocytosis, we frequently observe endocytic compartments of either type that are located closer to the back of the calyx than to the innervation side. This does not necessarily mean that the actual endocytosis event occurred at the back of the calyx. Instead, the compartment could have been trafficked to the backside. Moreover, such observations can be caused by the complex geometry of the calyx with its varying thickness and protrusions, which can hamper distance measurements between endocytic compartments and the innervation side. We thus also analyzed the distance to the back of the calyx to provide a more complete description of an endocytic compartment’s localization inside the calyx. The distance measurements toward both sides of the calyx were highly variable, even within single calyces, and did not show significant differences between Pitstop-2 treated calyces and control synapses (Table 1, nested one-way ANOVA). Although the cell-average based analysis of distances between the endocytic compartments and the PM did not yield significant results, changes in the distribution of the distance measurements could have been occluded in the analysis. We therefore performed a Q-Q-plot analysis which provides a sensitive way to detect such changes in measurement distributions between experimental conditions (Figure 5). The experimentally derived Q-Q-plots were compared to two simulated distributions assuming either identical (Figure 5, red lines) or similar, statistically not different, distributions (Figure 5, blue lines, see Materials and methods) between the experimental conditions. However, the Q-Q-plot analysis did not reveal any differences between individual distance distributions, since our data always resembled the case of a similar, not significantly different distribution (Figure 5, compare data points to blue lines).

**TABLE 1:** Distance measurements between endocytic structures and the PM at either the innervation side or the back of the synapse.

Experimental groups	HRP only	HRP+DMSO	Pitstop-2	Nested one-way ANOVA
Mean				
bSV- innervation side	518 ± 66	839 ± 216	644 ± 303	0.59
bSV- back of the synapse	183 ± 14	310 ± 59	221 ± 51	0.24
Large endosome-innervation side	183 ± 14	310 ± 59	221 ± 51	0.13
Large endosome-back of the synapse	115 ± 3	232 ± 43	179 ± 56	0.14

Cell-based average distances, given as mean ± SEM in nm.

**FIGURE 6:**
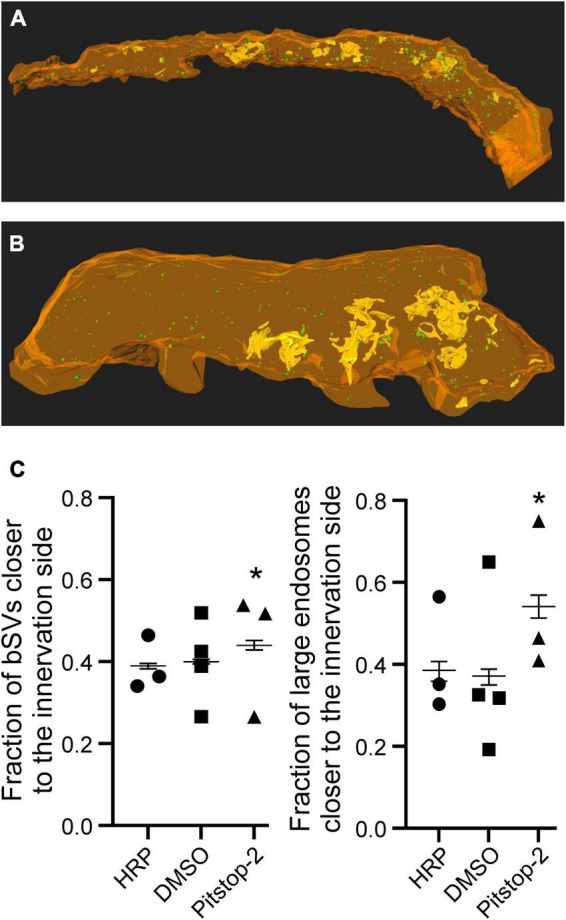
Application of Pitstop-2 alters the localization of large endosomes and bSVs at the calyx of Held. **(A)** 3D reconstruction of the endocytic compartments in the absence of Pitstop-2. **(B)** 3D reconstruction of the endocytic compartments in the presence of Pitstop-2. PM appears in orange, large endosomes are depicted in yellow and bSVs as green spheres. The innervation side is facing the bottom. **(C)** Quantification of the effects of Pitstop-2 on the localization of bSVs (left) and large endosomes (right) Please note that the weighted means are depicted. (*n* = 3–4 calyces/group, 1 calyx/animal; * = *p* < 0.05, Pitstop-2 different from both control groups).

In a final step, we considered the complex morphology of the calyx, especially its variable thickness and the presence of protrusions at the innervation side (see above) (Sätzler et al., 2002), and sorted the endocytic compartments into two categories: those being closer to the innervation side, i.e., the AZs, and those being closer to the back of the calyx. The sorting was based on whether the distance of a given endocytic compartment was shorter to the innervation side or the backside of the calyx and compensates for variability in distance measurement distributions due to variations in calyx morphology (Körber et al., 2012). Application of Pitstop-2 resulted in a shift of large endosomes toward the innervation side (Figure 6). The large endosomes were similarly distributed among the control groups (percentage of large endosomes closer to the innervation side: HRP only: 38.8 ± 4.1%, HRP and DMSO: 37.3 ± 3.9%), but the application of Pitstop-2 increased the fraction of large endosomes closer to the innervation side to 54.5 ± 4.9% (*d* = 0.64; *p* < 0.05 weighted mean analysis, Pitstop-2 different from both control groups) (Figure 6C). Additional to the effect of Pitstop-2 on the localization of large endosomes, it also altered the localization of bSVs, resulting in a mild shift of the bSV distribution toward the innervation side (percentage of bSVs closer to the innervation side: HRP only: 39.0 ± 1.2%, HRP and DMSO: 40 ± 1.1%, Pitstop-2: 44.1 ± 2.0%; *d* = 0.21; *p* < 0.05 weighted mean analysis, Pitstop-2 different from both control groups) (Figure 6C). Thus, impairing clathrin function by Pitstop-2 induced changes in the localization of endocytic structures, irrespective of the compartment type, at the calyx of Held *in vivo*. This suggests that clathrin may be important for different mechanisms of SV regeneration, CME and budding from large endosomes, under physiological activity patterns.

## Discussion

In the present study, we show that perturbing clathrin function results in the impairment of SV recycling at the calyx of Held synapse under physiological activity, *in vivo*. 3D EM analysis of endocytic structures revealed that application of the clathrin inhibitor Pitstop-2 impaired SV recycling via CME and/or budding from large endosomes. Our investigation of endocytic compartments relied on HRP, which is applied extracellularly and taken up into the presynapse by endocytosis. At the calyx of Held, we confirmed two types of HRP-labeled endocytic structures: bSVs and large endosomes (de Lange et al., 2003; Denker et al., 2011; Körber et al., 2012). bSVs are either created by CME or via budding from large, recycling endosomes. The large endosomes, however, can be generated by three distinct mechanisms: first, by the fusion of CME-derived vesicles either with each other or with early endosomes, second, by ADBE and third, by UFE (Richards et al., 2000; Rizzoli et al., 2006; Cheung et al., 2010; Hoopmann et al., 2010; Watanabe et al., 2013, 2014; Kokotos and Cousin, 2015). They are then either broken-up into SVs (Watanabe et al., 2014) or designated for lysosomal degradation, possibly after some time spend as a recycling endosome at the presynapse (Rizzoli, 2014).

In the auditory system, the physiological requirements on SV recycling change depending on the presence or absence of airborne sounds, as the former induces synaptic transmission at frequencies of more than 300 Hz for prolonged times (Kopp-Scheinpflug et al., 2008). The data presented here shows that clathrin is involved in the recycling of SVs and thus comprises one mechanism to meet the synaptic requirements of sound processing.

### Dynamics of large endosomes *in vivo*

Multiple mechanisms can lead to the formation of the large endosomes observed at calyx of Held *in vivo* (de Lange et al., 2003; Denker et al., 2011; Körber et al., 2012). Large endosomes are thought to endure for some time at the synapse, acting as sorting and/or recycling endosomes before they are transported back to the soma and follow the lysosomal route (reviewed in Rizzoli, 2014). Of note, small endosomes derived from UFE have been shown to degrade within seconds (Watanabe et al., 2013, 2014). The data shown here demonstrates that the application of Pitstop-2 affects large endosome volume and localization within the synapse without changing their density, implying that both parameters are depending on proper clathrin function. We observed a Pitstop-2-induced shift of large endosomes toward the innervation side of the calyx. This could imply that processes resulting in large endosome generation (see above) happen more often at the innervation side. However, an increase in endosome formation at the innervation side should have resulted in a concomitant increase in large endosome number and thus density in the calyces treated with Pitstop-2. We did not observe such an increase, but cannot formally exclude this possibility as small increases in endosome density could have been occluded by its relatively large variability. Moreover, the endosome density would have remained constant if an increase in endosome formation at the innervations side is accompanied by a decrease in endosome formation/transport at/to the back of the calyx. Nevertheless, a shift in endosome localization toward the innervation side is in line with a change to Pitstop-2-insensitive mechanisms of SV recycling which are expected to take place in proximity of AZs at the PM of the innervation (reviewed in Soykan et al., 2016; Chanaday et al., 2019).

In addition to the shift in localization, we observed an increase in the volume of large endosomes in synapses treated with Pitstop-2. This could be caused by a stronger employment of clathrin-independent endocytosis (CIE) modes such as UFE and ADBE and a consequential build-up of large endosomes, or by a decrease in clathrin-dependent SV budding from large endosomes. Both scenarios are in accordance with a dual role for clathrin in endocytosis and SV recycling at the calyx of Held *in vivo*.

### Regeneration of synaptic vesicles *in vivo*

Clathrin-mediated endocytosis (CME) has been regarded as the major mechanism of synaptic endocytosis during moderate synaptic activity. However, this view has been challenged by an increasing number of studies showing that CIE mechanisms play important roles under these conditions (reviewed in Soykan et al., 2017; Milosevic, 2018; Chanaday et al., 2019). Nevertheless, CIE mechanisms like UFE and ADBE likely require clathrin to dissolve the larger endosomes into SVs (Watanabe et al., 2014). Our results on bSV density and localization show that the application of Pitstop-2 reduced bSV density and shifted bSV localization toward the PM at the calyx of Held *in vivo*. Interestingly, we did not observe a reduction in the density of nl-SVs, probably due to the large number of SV at the calyx of Held (see Sätzler et al., 2002) and the relatively short incubation time of Pitstop-2 at the calyx (see below). However, the effects of Pitstop-2 application on the density and localization of bSVs suggest that different mechanisms of SV re-formation operate at the calyx, likely depending on the physiological demand on SV recycling in the presence or absence of sound perception.

The moderate reduction in bSV density observed upon Pitstop-2 application shows that at least a fraction of SV re-formation is clathrin-dependent. This could be either due to CME or clathrin-dependent SV budding from endosomes. Concomitant to the decrease in bSV density, we observed a shift in bSV localization toward the innervation side, suggesting that those SVs that are recycled in the presence of Pitstop-2 are located preferentially in a position favorable for future rounds of exocytosis. This preference is not surprising given the demand SVs for synaptic transmission during sound perception (de Lange et al., 2003; Borst and Soria van Hoeve, 2012; Körber et al., 2012). Of note, recently recycled SVs have also been shown to be randomly located in the presynaptic terminal of the neuromuscular junction (Rizzoli and Betz, 2004). However, the observed increase in large endosome volume upon Pitstop-2 treatment suggests that the reduction in bSV density is at least partially due to impairments in endosomal volume reduction by clathrin-dependent SV budding and that at least a part of the bSVs observed in the vincinity of the innervation side in the presence of Pitstop-2 is generated via CME.

Budding of SVs from endosomes is mechanistically different from CME as it requires AP-1 and/or AP-3 complexes instead of the AP-2 complex necessary for CME (Kim and Ryan, 2009; Glyvuk et al., 2010; Newell-Litwa et al., 2010; Kononenko et al., 2014). Thus, Pitstop-2 may preferentially impair AP-1/AP-3-dependent SV budding from large endosomes while AP-2-dependent CME prevails at least partially in the presence of Pitstop-2. However, we cannot rule out that bSVs were generated via CIE or another, yet unknown, Pitstop-2 insensitive budding mechanism. Interestingly, knock-down of the AP-2 complex in cultured hippocampal neurons slowed down endocytosis but did not fully block it (Kim and Ryan, 2009) suggesting alternative mechanisms of endocytosis operating at the PM. In line with this finding, intracellular application of Pitstop-1, a non-membrane permeable clathrin inhibitor (von Kleist et al., 2011), to the calyx also slowed down endocytosis without inhibiting it completely (Yue et al., 2017).

## Limitations and conclusion

Despite the effects of Pitstop-2 on SV recycling presented here, we would like to mention that the pharmacokinetics of Pitstop-2 *in vivo* are unknown. This poses a limitation to our approach as we cannot formally rule out that the concentration of Pitstop-2 at the synapse was too low to completely block clathrin-dependent processes. We therefore limited our analyses of Pitstop-2-induced effects to 30 min. after a single application. However, the fact that we were able to detect changes in the structure, density and localization of endocytic compartments after Pitstop-2 application implies that our approach interfered with clathrin function and resulted in an at least partial block of clathrin-dependent mechanisms. Nevertheless, long-term inhibition of clathrin by continuous application of Pitstop-2 (e.g., via an osmotic pump) and its effects on SV recycling and the usage of the reserve pool will be an interesting topic for future studies. Of note, two biochemical studies have raised doubts about the specificity of Pitstop-2 as a clathrin inhibitor (Dutta et al., 2012; Willox et al., 2014). However, there are also numerous studies showing Pitstop-2 to specifically block clathrin-dependent processes in neurons (von Kleist et al., 2011; Merriam et al., 2013; Neef et al., 2014; Missig et al., 2017; DiCello et al., 2019; Gomez et al., 2021; Zhang et al., 2021; Jaramillo-Polanco et al., 2022). Nevertheless, potential off target effects of Pitstop-2 need to be considered with respect to our results. There have been concerns regarding the proposed interaction sides of Pitstop-2 within the N-terminal domain of clathrin (Willox et al., 2014). Since we were not interested in the function of individual clathrin-domains, this concern does not affect the interpretation of our data as long as Pitstop-2 inhibits clathrin in some way. Moreover, it has also been reported that the application of Pitstop-2 prevented the clathrin-independent endocytosis of the major histocompatibility complex I (MHC I) (Dutta et al., 2012), which could interfere with our results. Clathrin-independent MHC I endocytosis is not well understood, but requires Arf6-GDP (Montealegre and van Endert, 2019). In neurons, Arf6 regulates the number of SVs and cisternal endosomes at the presynapse. Knock-down of Arf6 or its pharmacological inhibition result in a decrease in the number of SVs with a simultaneous increase in the number of docked SVs as well as the accumulation of cisternal endosomes in cultured hippocampal synapses (Tagliatti et al., 2016). If Pitstop-2 inhibits CIE via an Arf6-dependent mechanism, we should have observed the accumulation of cisternal/large endosomes at the calyx and/or a general reduction of the total number of SVs. Although we could have potentially missed a small reduction in SV number (see above), the increase in cisternal/large endosome number in hippocampal synapses is substantial and would have probably been detected in our experiments. Since we neither observed a reduction in total SV number nor an increase in the number of cisternal/large endosomes upon Pitstop-2 application, we conclude that a possible inhibition of Arf6-dependent CIE by Pitstop-2 does not interfere with our interpretation of the data presented. However, other Arf6-independent CIE processes include ADBE and UFE. The block of these two processes should have resulted in a decreased number of large endosomes, which we did not observe either. Thus, the present data obtained by our pharmacological approach likely allows us to draw valid conclusions about the nature of clathrin function during high frequency synaptic transmission *in vivo*.

The investigation of endocytic compartments by EM provides the best possible resolution but limits the investigation to a single time point per animal and thus precludes attempts to follow the fate of a given endocytic compartment over time. Further studies using, e.g., fluorescent endocytosis markers in combination with advanced imaging methods such as micro-endoscopy will shed light on such questions. Additionally, further studies that do not rely on the pharmacological inhibition of clathrin will be required to identify the precise nature of the clathrin-dependent mechanisms engaged in SV recycling *in vivo.* Lastly, our approach does not allow to discriminate SV recycling in response to sound processing from SV recycling under spontaneous activity. Although such a correlation would be highly desirable, it is hampered by the fact that hearing rats vocalize and it is thus very difficult to insulate them from sound perception. Correlations are further complicated by the high spontaneous activity of the calyx of Held even in the absence of sound perception (Kopp-Scheinpflug et al., 2008). Thus, even limiting analysis to calyces with high densities of endocytic compartment would not faithfully separate those with high spontaneous activities from those active due to sound processing.

Despite these limitations, we have shown that clathrin-dependent SV recycling from the PM and/or endosomal compartments accounts for at least a fraction of the regenerated SVs at the calyx of Held *in vivo*. Presumably, both pathways are active at the mature calyx in order to meet the high demand on SV recycling due to the processing of airborne sounds which requires faithful synaptic transmission at frequencies of several hundred Hertz for a prolonged time. We thus conclude that clathrin-mediated SV recycling is an integral part of the SV regeneration strategy at a central high frequency synapse *in vivo*.

## Data availability statement

The raw data supporting the conclusions of this article will be made available by the authors, without undue reservation.

## Ethics statement

The animal study was reviewed and approved by Regierungspräsidium Karlsruhe.

## Author contributions

CK conceived the project. AP, SH, YD, and HH performed the research. AP, KS, and CK analyzed the data. AP and CK wrote the manuscript with inputs from all authors. All authors contributed to the article and approved the submitted version.
